# Treatment of coronary pseudoaneurysm detected after percutaneous coronary intervention for chronic total occlusion: A case report

**DOI:** 10.1097/MD.0000000000032839

**Published:** 2023-03-03

**Authors:** Xudong Li, Yijie Huang, Lei Cui, Bing Han

**Affiliations:** a Department of Cardiology, Xuzhou Central Hospital, Jiangsu Province, China.

**Keywords:** case report, chronic total occlusion, coronary pseudoaneurysm, percutaneous coronary intervention, Polytetrafluoroethylene-coated stent

## Abstract

Coronary pseudoaneurysm (CPA) are associated with iatrogenic coronary artery dissection or perforation, which rarely reported formation early after percutaneous coronary intervention (PCI) for chronic total occlusion (CTO). This study reported a case of CPA that developed 4 weeks after PCI for CTO.

**Case report:**

A 40-year-old man was admitted with unstable angina and diagnosed with CTO of the left anterior descending artery (LAD) and right coronary artery. The CTO of the LAD was successfully treated by PCI. However, reexamination by coronary arteriography and optical coherence tomography after 4 weeks confirmed a CPA at the stented middle segment of the LAD. The CPA was treated surgically by the implantation of a Polytetrafluoroethylene-coated stent. reexamination at the 5-month follow-up revealed a patent stent in the LAD and no CPA-like manifestations. Intravascular ultrasound showed no intimal hyperplasia or in-stent thrombogenesis.

**Conclusion:**

CPA might develop within weeks after PCI for CTO. While it could be successfully treated by the implantation of a Polytetrafluoroethylene-coated stent.

## 1. Introduction

A coronary artery aneurysm (CAA) is a dilation of part of a coronary artery such that its diameter is at least 1.5-fold larger than that of the normal adjacent segments.^[1]^ The incidence of CAA after implantation of a second-generation drug-eluting stent is 1.02%, and CAA is associated with genetic predisposition, risk factors for coronary heart disease, and vascular wall injury or abnormalities.^[2]^ The pathogenesis of CAA involves coronary artery injury, intimal thickening and loss of elasticity, which lead to a gradual local protrusion of the vessel wall and formation of an aneurysm.^[3]^ A true aneurysm has an intact intima, media and adventitia, whereas a coronary pseudoaneurysm (CPA) lacks at least 1 of the arterial wall layers and consists of a single or double layer that protrudes outward. Although CPAs can form spontaneously, most cases occur following interventional therapy due to arterial wall injury caused by oversized balloons or stents, high-pressure balloon dilation, coronary atherectomy or laser angioplasty.^[4–6]^ This study reported an unusual case of a CPA that was detected 4 weeks after percutaneous coronary intervention (PCI) for chronic total occlusion (CTO).

## 2. Case report

A 40-year-old male patient was admitted to Xuzhou Central Hospital, Jiangsu, China on December 2020 with a 3-year history of episodic oppressive chest pain that had worsened during the previous month. The patient had a history of smoking for more than 20 years, hypertension for 4 years, type 2 diabetes mellitus for 10 years and hyperlipidemia for 4 years. There was no history of recurrent high fever, skin rashes, oral mucoconjunctivitis or lymph node enlargement, and Kawasaki disease. No family history of cardiomyopathy or sudden death was reported.

**Figure 1.**
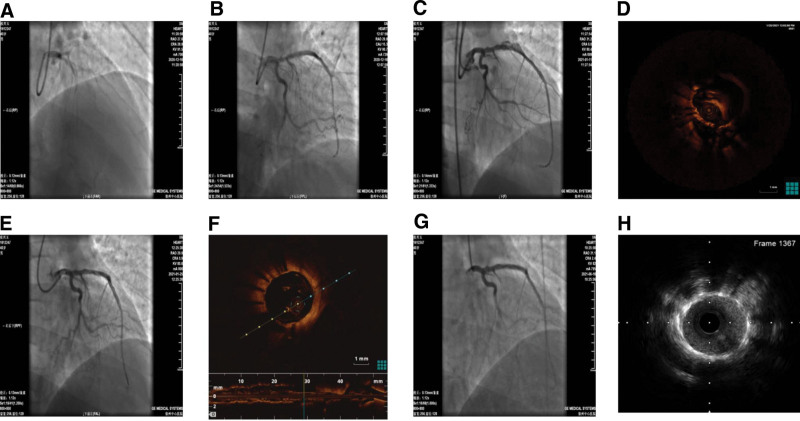
Images obtained before and after stent implantation. (A), CAG showed total occlusion of the middle segment of the LAD after the origin of D1. (B), CAG immediately after PCI. (C), CAG at 5 weeks after PCI revealed a fusiform aneurysm of the middle segment of the LAD. (D), OCT demonstrated interruption of the 3-layered structure of the wall of the middle segment of the stented LAD and outward protrusion of the adventitia. (E), CAG immediately after implantation of a coated stent in the middle segment of the LAD. (F), OCT showed complete closure of the aneurysm body and good adherence of the coated stent to the vascular wall. (G), CAG performed 5 months after implantation of the coated stent demonstrated a patent LAD with no evidence of restenosis in the stented middle segment. (H), IVUS performed 5 months after implantation of the coated stent showed no evidence of intimal hyperplasia or in-stent thrombogenesis. CAG = coronary arteriography, IVUS = intravascular ultrasound, LAD = left anterior descending artery, OCT = optical coherence tomography, PCI = percutaneous coronary intervention.

Physical examination on admission revealed that the patient had a blood pressure of 130/70 mm Hg in the left upper limb and 140/72 mm Hg in the right upper limb. The patient was in normal sinus rhythm and physiological heart murmurs. There were no dry or moist rales in the lungs or edema of the lower limbs. The electrocardiogram showed that the patient was in sinus rhythm on admission. Echocardiography revealed a left ventricular end-diastolic diameter of 51 mm and an ejection fraction of 58%. The results of laboratory investigations were normal except low-density lipoprotein cholesterol: 3.67 mmol/L (reference range: 2.20–3.60 mmol/L). coronary arteriography (CAG) showed no stenosis of the left main coronary artery or left circumflex artery, total occlusion of the middle segment of the left anterior descending artery (LAD) distal to the origin of the first diagonal artery (Fig. 1A), and total occlusion of the proximal right coronary artery with a bridging lateral branch between the segments proximal and distal to the occlusion. The patient was diagnosed with atherosclerotic coronary heart disease and unstable angina.

The occlusion of the LAD was treated by percutaneous coronary intervention (PCI). Initial attempts to deliver Fielder XT-R, Gaia 2 or Gaia 3 guidewires (Asahi Intecc, JPN) through the occluded segment of the LAD failed. Subsequently, an antegrade ConquestPro guidewire (Asahi Intecc, JPN) was inserted successfully to open the occluded segment, and visualization of the microcatheter confirmed that the distal end of the guidewire was in the distal true lumen of the LAD. Tandem implantation of 2 Firehawk drug-eluting stents (Shanghai Microport, China) between the middle-distal segment and proximal segment of the LAD was performed (Fig. 1B). PCI of the right coronary artery was scheduled for 5 weeks later.

reexamination by CAG on January 11, 2021 showed irregular fusiform ectasia of the middle segment of the stented LAD, and the base of the aneurysm was relatively wide and connected to the true lumen (Fig. 1C). Further examination by optical coherence tomography (OCT) showed loss of vascular wall integrity in the aneurysm with interruption of the 3 layers of the vascular wall and outward protrusion of the adventitial layer (Fig. 1D), suggesting that a CPA had formed. The CPA was treated surgically by the implantation of a Polytetrafluoroethylene (PTFE)-coated stent. A 3.5 mm × 26 mm Graft master coated stent (Abbott Vascular, USA) was placed and deployed at 16 atm in the middle segment of the LAD under OCT guidance to cover the aneurysm opening, and QuantumNC balloons (3.5 mm × 15 mm deployed at 16 atm, and 4.0 mm × 12 mm deployed at 12 atm) were used for postdilation. Subsequent CAG showed that the body of the aneurysm had disappeared (Fig. 1E). reexamination by OCT showed that the pseudoaneurysm was covered and that the Graft master stent was expanded and well adhered to the vessel wall (Fig. 1F). The patient was given maintenance dual antiplatelet therapy (aspirin 100 mg qd and ticagrelor 90 mg bid) postoperatively.

No adverse cardiovascular events occurred during the 5-month follow-up. reexamination by CAG showed a patent stent in the LAD and no signs of coronary artery ectasia (Fig. 1G). Intravascular ultrasound performed 5 months after implantation of the coated stent demonstrated good adhesion of the stent in the LAD and no evidence of intimal hyperplasia, restenosis or in-stent thrombosis (Fig. 1H).

## 3. Discussion

This study reported that the CPA in this case was successfully treated by the implantation of a PTFE-coated stent. The features of this unusual case may provide diagnosis and treatment experience for clinicians.

Complex cases of CTO are generally managed using antegrade, retrograde or hybrid techniques. Although these techniques improve the success rate of PCI for complex cases of CTO, they can damage the elastic component of the media layer and predispose to gradual enlargement of the lumen (particularly when high-pressure dilation is used after stenting), vascular wall thinning and remodeling, and formation of a CPA. The reported case had no history of Kawasaki disease, speculate that CPA may have arisen as a consequence of coronary artery wall injury induced by guidewire escalation and dissection caused by high-pressure balloon dilation.

Interventional therapy with a coated stent is particularly suitable for young patients with an aneurysm of a single coronary artery branch and normal left ventricular function, the successful use of this technique in patients with CPA has been reported previously.^[7,8]^ The case in this report was treated by the implantation of a coated stent, and OCT was used to identify the CPA opening and determine the optimal landing position for the coated stent. PTFE-coated stents can help to prevent plaque prolapse, restore vascular wall continuity and reduce the impact of blood flow on plaques. Furthermore, the negative charges on PTFE are thought to inhibit protein agglutination on the tissue surface and thereby reduce platelet activation and thrombosis. Additionally, the neointima on the stent surface may facilitate sealing of the aneurysm by reducing blood flow into the CPA and promoting thrombosis. Although coated stents are associated with risks of branch loss, in-stent restenosis and in-stent thrombosis, a recent study reported that coated stent implantation had an acceptable safety profile with a relatively low incidence of adverse cardiac events.^[9]^ In the present case, the 5-month follow-up showed that the aneurysm lumen was closed, the stent was patent, and no in-stent restenosis or thrombosis had occurred.

It is important that clinicians are aware of the risk of iatrogenic CPA. PCI for complex cases of CTO (especially after failure of previous interventional therapy) may involve guidewire changes and escalation that promote expansion of a false lumen and hematoma formation. The use of preoperative coronary computed tomographic angiography and intraoperative intravascular ultrasound may.

help to improve the success rate of PCI for anatomically complex CTO (such as abnormal or occluded vessel origin, absence of a proximal stump, severe tortuosity or long occluded segment with an unclear course) and reduce the occurrence of CPA. antiplatelet therapy and reduction of cardiovascular risk factors also play important roles after intervention for CPA. In conclusion, the CPA in this case was successfully treated by the implantation of a PTFE-coated stent. Nevertheless, clinicians should be aware of the risk of in-stent restenosis/thrombosis during follow-up.

## Author contributions

**Conceptualization:** Yijie Huang, Lei Cui.

**Formal analysis:** Lei Cui.

**Supervision:** Yijie Huang, Bing Han.

**Writing – original draft:** Xudong Li.

**Writing – review & editing:** Xudong Li.
